# Correction: Conversion of *N*-acyl amidines to amidoximes: a convenient synthetic approach to molnupiravir (EIDD-2801) from ribose

**DOI:** 10.1039/d2ra90030k

**Published:** 2022-04-04

**Authors:** Ajaz Ahmed, Qazi Naveed Ahmed, Debaraj Mukherjee

**Affiliations:** Natural Product and Medicinal Chemistry Division, Indian Institute of Integrative Medicine (IIIM) Jammu 180001 India dmukherjee@iiim.ac.in dmukherje@iiim.res.in; Academy of Scientific and Innovative Research (AcSIR) Ghaziabad-201002 India

## Abstract

Correction for ‘Conversion of *N*-acyl amidines to amidoximes: a convenient synthetic approach to molnupiravir (EIDD-2801) from ribose’ by Ajaz Ahmed *et al.*, *RSC Adv.*, 2021, **11**, 36143–36147, DOI: 10.1039/D1RA06912H.

The authors regret that affiliation *b* was incorrectly given in the original article. The corrected affiliation is shown here.

The Royal Society of Chemistry apologises for these errors and any consequent inconvenience to authors and readers.

## Supplementary Material

